# Genito-Urinary Surgery

**Published:** 1904-09-03

**Authors:** 


*(To be continued.)*

### GENITO-URINARY SURGERY.

**Cryoscopy.**—Koranyi,[1] an authority on the subject, has written a paper dealing with the principles upon which this method of examining fluids by the determination of their freezing-points depends. ("Moderne ärtz. Bibliothek," Heft. I, 1904).

According to Van't Hoff's theory of solutions any substance in solution behaves like gas in a closed vessel and tends to occupy as large a space as possible and thus exerts osmotic pressure. This pressure can be measured and is found to depend on the nature of the dissolved substance, its concentration and its temperature. Solutions of equal osmotic pressure, *i.e.*, isotonic solutions, contain in equal volumes an equal number of molecules and have the same freezing-point. The freezing-point of a solution is lowered in proportion to the number of molecules in a unit of volume of the fluid. The metabolism of nitrogenous compounds in the body is a constant source of increased osmotic pressure owing to the number of dissolved molecules produced thereby. The kidneys regulate the osmotic pressure by removing superfluous molecules and water; they have a concentrating and a diluting power, and diseases may affect one or the other power, or both. Hence, after removal of the kidneys, osmotic pressure rises from the accumulation of organic molecules. Relatively more molecules than water have to be excreted, and hence usually the freezing-point of the urine is lower than that of the blood. Inferences from the depression of the freezing-point of urine alone are apt to be fallacious, but if much water be drunk, valuable information may be obtained. Weakened kidneys can excrete neither highly concentrated nor very dilute urine, so that the freezing-point is not diminished as it should be by drinking copiously of fluids. In parenchymatous nephritis both the concentrating and the diluting powers of the kidneys are lost. There is increased thirst and greater intake of fluid, but the freezing-points of the blood and urine are unaltered and dropsy ensues. Theodore Ticken [2] has examined the blood and urine in 330 cases and confirms the results of other observers as to the facts of cryoscopy and its clinical usefulness.

**Renal Decapsulation.**—A. G. Grunwell [3] reports a case in which renal decapsulation proved very beneficial. The patient, a man, had suffered from great pain in the region of the left kidney and passing to the testicle, for four months. Chloroform had to be given to relieve paroxysms of severe pain several times. The urine only contained albumen in traces at times, and there was no hæmaturia. The kidney on exposure was found to be large, congested, and friable. No stone was found in the renal pelvis. The decapsulation was performed the following day. At the end of three months the patient returned to duty free from pain and albumen. The decapsulation was not performed at the first operation because the nephrotomy had produced hæmorrhage and shock. The author regards the intense venous engorgement of the kidney as due to an early stage of chronic nephritis. J. Walker Hall and G. Herxheimer [4] record some experimental work carried out in an investigation of the effects of decapsulation of the kidneys in certain animals. They found, as others had, that when the capsule was removed from the normal kidneys of rabbits another capsule was formed, which at the end of 10 to 21 days was represented by a fibrous layer thicker than the original capsule; but in 35 of the animals examined they did not find any marked formation of new blood-channels between the kidney and the adherent tissues. Edebohls considers that in cases of nephritis in man such blood-channels form after decapsulation and increase the nutrition of the kidney, causing extensive regeneration of the secreting cells in nephritis. The authors then produced nephritis in rabbits by injecting neutral ammonium chromate; and subsequently, at various intervals, performed decapsulation, and, later still, examined the kidneys after killing the animals. This experimental work seems to show that decapsulation does not lead to increased vascular anastomosis between the vessels in the kidney and those in the surrounding tissues. In fact, decapsulation seemed rather to accelerate interstitial inflammation. It is suggested that in acute nephritis, anuria, and hæmaturia the operation gives relief by diminishing tension, and might be resorted to earlier for post-febrile and acute renal manifestations. It is pointed out that Bonez-Osmolowsky similarly failed to find in rabbits any new formation of anastomotic blood-vessels after decapsulation. Leonard Freeman [5] reports a case in which copious and prolonged renal hæmorrhage was cured by decapsulation of one kidney. The patient was a man of 59, who was anæmic and emaciated, and gave a history of liability to occasional attacks of hæmaturia for 20 years. For a year before the operation the bleeding had been constant and profuse. Medical treatment had proved unavailing. With the Harris segregator the bleeding was shown to come only from the left kidney. A diagnosis of hæmorrhage from chronic interstitial nephritis of the left kidney was made and decapsulation of that organ performed. Considerable hæmorrhage from the cortex occurred. After the operation the blood rapidly vanished from the urine. A small piece of the renal cortex, excised at the operation, showed glomerulo-nephritis on microscopical examination. Eight months later the patient was in excellent condition, and the urine contained no albumen and no casts, and had a specific gravity of 1022. For this form of renal hæmorrhage decapsulation appears to be the best operation, for it checks both the hæmorrhage and the nephritis which causes the hæmorrhage.

**Movable Kidney.**—C. D. Aaron [6] has written a paper on "Treatment in 442 cases of Movable Kidney without Surgical Intervention." He thinks operation should be performed only when proper mechanical support fails to hold the kidney in position. Operation yields a mortality of 2 to 5 per cent., often a second operation is required owing to the failure of the first, and gastro-intestinal and nervous symptoms are not always removed by operation. The author believes that 90 per cent. of the patients can be relieved without operation. Movable kidney may lead to constipation, backache, debility, acne, headache, nervous exhaustion, and many other symptoms. Other abdominal organs are often simultaneously prolapsed, and they must be held in position before a cure can be effected. No pressure should be exerted upon the kidney itself, but a proper band should be applied over the whole lower abdomen to cause a uniform pressure there without pressure upon the upper abdomen. The supporting band is worn for one year.

[1] Abst. Edin. Med. Jour., April 1904, p. 359. [2] Med. Record, March 12, 1904, p. 437. [3] Ibid, March 26, 1904, p. 484. [4] Brit. Med. Jour., April 9, 1904, p. 819. [5] Annals of Surgery, March 1904, p. 370. [6] Abst. Treatment, March 1904, p. 31.

